# How were situations of preventive and curative care expenditure for AIDS and medical burden of patients? Research based on "System of Health Accounts 2011"

**DOI:** 10.1186/s12889-019-8131-0

**Published:** 2020-02-04

**Authors:** Huan Zhan, Qiong Wu, Shuang Zang, Liangrong Zhou, Xin Wang

**Affiliations:** 1 School of Humanities and Management, Hunan University of Chinese Medicine, 300 Xueshi Road, Yuelu District, Changsha, Hunan Province 410208 People’s Republic of China; 2 School of Nursing, China Medical University, Shenyang, Liaoning Province People’s Republic of China; 3 Humanity and Management College, Shaanxi University of Chinese Medicine, Xianyang, Shaanxi People’s Republic of China; 4 College of the Humanities and Social Sciences, China Medical University, Shenyang, Liaoning Province People’s Republic of China

**Keywords:** AIDS, SHA2011;preventive care expenditure;curative care expenditure;Total expenses of hospitalization;out-of-pocket payments

## Abstract

**Background:**

The problem of AIDS response has not only involved public health, but also had a great impact on the family burden.The objective of this study was to estimate the preventive and curative care expenditure(PCE)for AIDS of Hunan Province in 2017 based on System of Health Accounts 2011(SHA2011)by quantity,financing scheme,health provider,health function,and to analyses the factors affecting patients’ medical burden.

**Methods:**

Through stratified multi-stage sampling method, 1336 institutions were surveyed to obtain AIDS prevention and control data, and the official data collected from Health Statistical Yearbook, Health Financial Annual Reports and Government Input Monitoring System were used to estimate the AIDS PCE based on SHA2011. Univariate analyses and ordered logistic regression were used to evaluate the factors affecting the medical burden of AIDS patients.

**Results:**

The AIDS PCE of Hunan Province in 2017 was 266.67 million, mainly flowed to hospitals and disease prevention and control institutions. The proportions of curative care expenditure(CCE) and prevention expenditure were 51.39 and 48.61% respectively. Prevention expenditure were mainly used for traditional prevention methods. All prevention expenditure and 88.52% of CCE were borne by public financing scheme. Family health expenditure accounted for 11.12% of CCE, but there were still some people with heavy burden of treatment. Non insurance, co-infection and length of stay are risk factors to the total hospitalization expenses(Totalexp)and the out-of-pocket payments(OOPs)(all *p* < 0.05,*OR* > 1). Taking the age group under 30 as the reference, the partial regression coefficient of the age group over 60 was statistically significant (*OR*_(Totalexp)_ = 1.809, *OR*_(OOPs)_ = 0.30).

**Conclusion:**

The financing structure of the PCE for AIDS in Hunan Province was relatively stable and the flow of institutions was reasonable. The functional flow of expenditure embodied the principle of “prevention first”. China should incorporate oral PrEP into the national guidelines as soon as possible to improve the allocation efficiency of AIDS prevention resources. Meantime, several measures should be taken to reduce the medical burden of AIDS patients, including expanding the scope of government assistance, adjusting insurance compensation measures, increasing the rate of patients participating in insurance,encouraging commercial insurance to join the AIDS insurance system,and controlling length of stay in hospital.

## Background

AIDS is a malignant infectious disease caused by the human immunodeficiency virus (HIV). Up to now, about 37 million people are living with HIV all over the world [1]. In China, the number of AIDS cases has reached 850,000. According to the Statutory Infectious Diseases Report in China (2017), 77.16% died of AIDS [2]. The AIDS response has not only involved public health, but also exerted a great impact on the family burden [3]. It is of great significance to study the preventive and curative care expenditure (PCE) of AIDS for the evaluation of the rational allocation of AIDS resources.

Some countries established National AIDS Account(NAA) by using the policy analysis framework of the National Health Accounts (NHA) [4]. NHA model can be used to analyze the financing sources, institutional flows and functional allocation of PCE for AIDS [5], and provide valuable information to optimize the collection and allocation of AIDS resources [6]. In 1999, data on the use of HIV services collected in Rwanda under the framework of NHA showed that 93% of funds were provided by households, about 6% by donors and less than 1% by the government [7]. Fortunately, in recent years, great changes have taken place in the financing structure of AIDS prevention and control in Rwanda. There are community health workers to provide free AIDS prevention services for citizens. Through the implementation of micro insurance scheme, the proportion of out-of-pocket payments (OOPs) of insured patients has decreased to 10%. By mobilizing domestic and international funding, the poorest 25% are exempt from payments for insurance and OOPs [8]. The NHA-based results in Thailand demonstrated that 81% of the funds come from public financing, and about 2% was paid by households in 2003 [9]. Since 2015, the Ministry of Public Health has increased its budget for a strategy to eliminate AIDS [10].

In addition, the Joint United Nations Programme on HIV and AIDS(UNAIDS) exploited and initiated the National AIDS Spending Assessment(NASA) in 2006 to track actual expenditure on national or regional AIDS resources. A number of NASA-based studies have found that countries spend the most on prevention, with China, Nepal, and Sierra Leone accounting for 54.31, 46, and 61%, respectively [11, 12]. According to Thailand’s 2013 NASA, 89% of all funding for HIV that year went to treatment, support and clinical care. In contrast, only 3.6% of expenditures were devoted to HIV prevention, detection and counselling for the susceptible population [13].

Whether based on NHA or NASA, we can calculate the financing structure and flow direction of AIDS prevention and control fund, but we can not specify the individual burden of different groups of AIDS patients. Studies on various kinds of AIDS patients’ OOPs have been carried out through social survey. A research for OOPs incurred by individuals with HIV in Dominica showed that the OOPs exhibited regressivity, with lower-wage patients spending proportionally more to receive care [14]. In Canada, a descriptive study of the potential costs of HIV infected people found that patients’ OOPs accounted for ranging from 0 to over 50% of antiretroviral cost when there were inter- and intra-jurisdiction heterogeneity in antiretroviral drug cost sharing policies [15]. Patients with HIV in China can get free antiretroviral treatment relying on the financial support of the government [16]. But if they suffers from other diseases or needs to receive other health care services, part of the medical costs will be borne by the family. This study conducts subnational PCE for AIDS based on the System of Health Accounts 2011(SHA2011). It can not only better explain the quantity,financing scheme,health provider, health function of PCE by the matrix cross analysis, but also subdivide the curative care expenditure(CCE) by age to reflect the health expenditure burden of different groups. Besides, we also intend to find out the influencing factors of AIDS patients’ treatment burden by analyse survey data.

## Methods

### Data source

The basic data used in this study were obtained from the current health expenditure research project in Hunan Province. Data were divided into two categories. A class of data were collected from official statistic data of Health Commission of Hunan Province, such as *Health Statistics Yearbook*, *Health Financial Yearbook*, Government Input Monitoring Data of Hunan Province in 2017, etc. The other was procured from the sample institutions.

### Study sample

In this study, the multi-stage stratified sampling method was used to determine the sample institutions. In the first stage, Changsha, Yueyang, Zhuzhou, Yongzhou and Hengyang were selected as sample areas based on the comprehensive consideration of the economic development, demographic characteristics, medical and health conditions, and cultural differences. The second stage was to select one district and three counties from the monitoring cities. The third stage was to choose medical and public health institutions in the monitoring areas at all levels according to the type of institutions of administrations structure. The sample data were obtained from 76 hospitals, 28 public health institutions, 36 community health service centers, 102 township hospitals, 720 village clinics and 168 private clinics.

The survey data included the income and expenditure of AIDS prevention and treatment services carried out by the sample institutions throughout 2017. Preventive data were obtained from the questionnaires completed by sample institutions, involving the information of human input, income, expenditure and special subsidy for AIDS prevention. The curative care data were derived from the information system of the sample institutions, involving age, gender, disease, length of stay, total and detailed costs of treatment, types of insurance coverage, insurance reimbursement, and OOPs of patients, but did not contain any information that may infer personal identity. After the invalid or false information was excluded in this study, there were 10,335,678 items involving the treatment and nursing expenditure, of which 1018 items of AIDS treatment were included. The sample of AIDS data were collected from 196 outpatients and 822 inpatients from 0 to 85 years old (770 male and 248 female involved).

### Statistical method

Data were recorded and sorted with SPSS 17.0 and STATA 12.0 were used to calculate the PCE for AIDS.

### Calculation of PCE for AIDS

The PCE of AIDS based on SHA2011 framework refers to the monetary performance of residents’ AIDS prevention and curative service consumption consumed by a country (region) in a certain period (usually one year), excluding capital investment such as fixed assets construction. The preventive care is limited to primary and secondary prevention in SHA 2011, so the expense of antiretroviral drugs for HIV-infected people is excluded in prevention expenditure, but it is included in the CCE in our study. The PEC formula for AIDS can be found in “A System of Health Accounts 2011 Edition” [17].

### Influencing factors of hospitalization expense and out-of-pocket expenditure for AIDS patients

Over 90% of CCE of AIDS was spent on inpatient services. Taking the hospitalization cost as the object, the total hospitalization expense(Totalexp) and OOPs were studied on AIDS patients with different gender, age, length of stay, insurance plan and opportunistic infection. For that the inpatient data were distributed unnormally, Mann-Whitney U test was performed on the data among the two groups, and Kruskal-Wallis H test was carried out on the data between the multiple groups. Spearman correlation analysis was conducted to determine the relationship between length of stay and expenditure. Based on the results of univariate analysis, variables were selected for ordered logistic regression analysis to evaluate the factors affecting the Totalexp and OOPs(α = 0.05).

## Results

### Fundamental result of PCE for AIDS

In 2017, the PCE for AIDS in Hunan Province was 266.67 million RMB, accounting for 5.29% of the expenditure of prevention and treatment of infectious disease.

### Functional distribution of PCE for AIDS

From the functional distribution of PCE, 51.39% was used for prevention and 48.61% for treatment. As for the treatment expense, 45.24% (58.66 million RMB) expenditure was spent on free antiretroviral drugs for HIV-infected patients provided by the government, 54.76% was used to pay for other health care services (including treatment of opportunistic infections) for AIDS patients. The expenditure of preventive care was mainly applied to education of AIDS prevention, free distribution of condoms, the purchase of HIV testing reagents and HIV testing services, HIV mother-to-child transmission testing, free screening of pregnant women, etc.

### Allocation of medical institution in PCE for AIDS

52.56% of the PCE for AIDS occurred in the general hospitals, and 26.47% went to disease prevention and control institutions. Maternal and child health care hospitals occupied 13.00%. Blood collection and supply institutions accounted for 6.52%. While the expenditure for other institutions was less than 2.00%. The expenditure of prevention mainly went to the institutions of disease control and prevention (51.52%). Almost all curative care expenditure went to general hospitals(99.64%) (Table 1).

**Table 1:** Allocation of medical institution in PCE for AIDS

	The total Expenditure		Preventive care expenditure		Curative care expenditure	
	(million)	%	(million)	%	(million)	%
General hospital	140.15	52.56	10.98	7.84	129.17	92.16
Special hospital	0.42	0.16	0.08	18.68	0.34	81.32
Traditional Chinese medicine hospital	0.90	0.34	0.77	85.57	0.13	14.43
Maternity and child care hospital	34.66	13.00	34.66	100.00	0.00	0.00
Primary medical institutions	0.40	0.15	0.40	100.00	0.00	0.00
Disease prevention and control institutions	70.59	26.47	70.59	100.00	0.00	0.00
Blood collection and supply institutions	17.40	6.52	17.40	100.00	0.00	0.00
Rehab	0.14	0.05	0.14	100.00	0.00	0.00
Health supervision office	0.07	0.02	0.07	100.00	0.00	0.00
Administrative units	1.95	0.73	1.95	100.00	0.00	0.00
Total	266.67	100.00	137.03	51.39	129.64	48.61

### Financing scheme in PCE for AIDS

As shown in Table 2, the PCE for AIDS was dominated by public financing, accounting for 94.42%, among which government financing and social health insurance took for 84.17 and 10.25%, respectively. Household health expenditure accounted for 5.41%, and the voluntary financing was about 460,000 RMB, a extremely small proportion of the total expenditure. In terms of the financing structure of different functional expenditure, all prevention services and 88.52% of curative care were borne by public financing.

**Table 2:** The composition of financing programs for AIDS in 2017

	The total Expenditure		Preventive care expenditure		Curative care expenditure	
	million	%	million	%	million	%
Public financing scheme	251.78	94.42	137.03	100.00	114.75	88.52
Government financing scheme	224.44	84.17	137.03	100.00	87.41	67.43
Social health insurance	27.34	10.25	0.00	0.00	27.34	21.09
Mandatory medical savings account	0.46	0.17	0.00	0.00	0.46	0.35
Family health expenditure	14.42	5.41	0.00	0.00	14.42	11.12
Total	266.66	100.00	137.03	100.00	129.63	100.00

### CCE of different age groups

By the end of 2017, a total of 13*,*636 AIDS patients had survived in Hunan Province. The antiretroviral treatment for HIV of medical institution are free, but if they provide other treatment there is a fee. The per capita treatment expenditure (excluding the cost of antiretroviral treatment borne by the government) of AIDS patients was 8415.22 RMB, which was higher than that of the whole population in this area in that year. From the perspective of the family treatment costs of patients in all age(Table 3), the proportion of family health expenditure of AIDS patients aged 1–19 and 30–49 y was over 30%, and the patients aged 40–49 y even exceeded 60%. According to the standards of World Health Report [18], these people are at the risk of poverty.

**Table 3:** CCE of different age groups (%)

Age groups	Public financing scheme			Mandatory medical savings account	Family health expenditure
	Total	Government financing scheme	Social health insurance		
0–9	64.06	8.99	55.08	0.00	35.94
10–19	52.83	5.86	46.97	0.14	47.03
20–29	72.19	72.19	0.00	0.07	27.74
30–39	50.67	23.72	26.95	0.90	48.43
40–49	38.89	11.12	27.77	0.99	60.12
50–59	83.82	38.22	45.60	0.94	15.24
60–69	89.15	41.84	47.31	0.94	9.91
70–79	90.69	52.28	38.42	0.00	9.31
80+	88.78	53.31	35.46	0.00	11.22

### Influencing factors of Totalexp and OOPs

To seek out the factors about the family burden, the survey data of 822 inpatients were statistically analyzed. According to the results of nonparametric test (Table 4)and analysis of Spearman correlation (Table 5), excluding gender (*P* = 0.547 > 0.05), the differences of Totalexp(not including the cost of antiretroviral treatment) among groups of other factors had statistically significance(*P* < 0.001). There was no significant correlation between gender and OOPs(*P* = 0.288 > 0.05). The age, length of stay, insurance and opportunistic infection were correlated with OOPs, and the differences were statistically significant (*P* < 0.001).

**Table 4:** Univariate analysis of influence on the Totalexp and OOPs of AIDS patients

Variable	*N* = 822	Totalexp (RMB)			OOPs (RMB)		
		Median(P_25,_ P_75_)	U / H	*p*	Median(P_25,_ P_75_)	U / H	*p*
Sex							
Female	177	4542.32 (3749.02, 5816.17)	55,397.00^a^	0.547*	1103.56(0.00,1592.27)	54,224.00^┼^	0.288*
Male	645	4639.03(3747.16,5868.86)			1010.04(0.00,1476.34)		
Age							
< 30 years	31	4838.83(3307.98, 16,946.80)	10.22^b^	0.037**	1522.91(621.98,16,946.90)	69.92^b^	< 0.001**
31-40 years	71	4155.90 (2932.93, 7905.78)			1294.06 (0.00,3934.60)		
41-50 years	168	4494.78 (3613.57,6791.47)			1183.07(0.00,3358.10)		
51-60 years	158	4430.08 (3576.15, 5253.15)			1174.54(0.00,1512.47)		
> 60 years	396	4773.16 (4000.53,5648.70)			0.00(0.00,1324.345)		
Insurance							
Urban employee insurance	123	4460.77(3608.78, 5255.86)	114.53^b^	< 0.001**	0.00(0.00,0.00)	238.91^b^	< 0.001**
Insurance for urban and rural residents	626	4537.26(3725.67, 5478.605)			1071.33 (0.00,1414.52)		
Self-paying	73	24,524.5(11,666.15,28,056.65)			24,524.5(11,666.15,28,056.65)		
Opportunistic infection							
No	730	4498.83(3696.45,5367.01)	7983.00^┼^	< 0.001*	740.24 (0.00,1367.60)	1835.50^┼^	< 0.001*
Yes	92	22,704.65(8787.97,27,747.97)			21,867.65 (4734.08,27,670.20)		

^a^ Mann-Whitney U of Mann-Whitney U test

^b^ Chi-Square of Kruskal-Wallis H test

* *p*-value of Mann-Whitney U test

** *p*-value of Kruskal-Wallis H test

Note: 346 patients in the survey received medical assistance from the government in addition to insurance reimbursement, and their OOPs was 0. Among them, 95 patients were insured by urban workers’insurance and 251 were insured by urban and rural residents’ insurance

**Table 5:** Spearman correlation analysis of length of stay between the Totalexp and OOPs of AIDS patients

	Totalexp (RMB)		OOPs (RMB)	
	***r***	***p***	***r***	***p***
Length of stay	0.65	< 0.001	0.213	< 0.001

Table 6 & 7 showed the results of ordered logistic regression analysis about Totalexp and OOPs. Taking the group under 30 as the reference, the partial regression coefficient of the age group over 60 was statistically significant, *OR*_(Totalexp)_ = 1.809, *OR*_(OOPs)_ = 0.30. It was found that compared with the group under 30, the age group over 60 had higher hospitalization costs and lower OOPs. Taking self-paying patients as the control group, the partial regression coefficients of urban employee insurance and urban and rural residents groups were statistically significant, *OR*_(Totalexp)_ = 0.239/0.158, *OR*_(OOPs)=_0.0.01/0.13, indicating that urban employee insurance and urban and rural residents groups were lower than that of self-paying patients either on Totalexp or OOPs. Taking the group of none opportunistic infection as the reference, the partial regression coefficient of the group with opportunistic infection was statistically significant, *OR*_(Totalexp)_ = 11.056, *OR*_(OOPs)_ = 75.49. The partial regression coefficient of length of stays was statistically significant, *OR*_(Totalexp)_ = 1.41, *OR*_(OOPs)_ = 1.06.

**Table 6:** Ordered logistic regression analysis affecting the Totalexp

	b	*S*_*b*_	*Wald χ*^*2*^	*P*	*OR*	*OR 95% CI*	
Age							
> 60 years	0.903	0.418	4.677	0.031	2.467	1.089	5.596
51-60 years	0.343	0.426	0.65	0.42	1.409	0.612	3.245
41-50 years	0.018	0.427	0.002	0.966	1.018	0.440	2.354
31-40 years	−0.46	0.48	0.918	0.338	0.631	0.247	1.618
< 30 years					1		
Insurance							
Urban employee insurance	−1.43	0.577	6.148	0.013	0.239	0.077	0.741
Insurance for urban and rural residents	−1.846	0.563	10.769	0.001	0.158	0.052	0.475
Self-paying					1		
Opportunistic infection							
No					1		
Yes	2.403	0.429	31.42	< 0.001	11.056	4.773	25.636
Length of stay	0.342	0.025	192.423	< 0.001	1.41	1.34	1.48

**Table 7:** Ordered logistic regression analysis affecting OOPs of AIDS patients

	*b*	*S*_*b*_	*Wald χ*^*2*^	*P*	*OR*	*OR 95% CI*	
Age							
> 60 years	−1.214	0.427	8.072	0.004	0.30	0.13	0.69
51-60 years	−0.513	0.437	1.38	0.24	0.60	0.25	1.41
41-50 years	− 0.031	0.444	0.005	0.944	0.97	0.41	2.31
31-40 years	−0.069	0.501	0.019	0.891	0.93	0.35	2.49
< 30 years					1		
Insurance							
Urban employee insurance	−4.702	0.864	29.627	< 0.001	0.01	0.00	0.05
Insurance for urban and rural residents	−2.044	0.834	6.008	0.014	0.13	0.03	0.66
Self-paying					1		
Opportunistic infection							
No					1		
Yes	4.324	0.686	39.75	< 0.001	75.49	19.69	289.74
Length of stay	0.055	0.017	9.966	0.002	1.06	1.02	1.09

## Discussion

At present, there is no universal standard to measure the scale of the cost of AIDS prevention and treatment. In the international community, the PCE of AIDS is often discussed from the perspective of financing structure. In recent years, China’s government has continuously increased its investment in the prevention and control of AIDS. In 2017, China’s fiscal expenditure was almost 7 billion RMB on its AIDS response, an increase of more than 5.4% from 2016 [19]. Taking Hunan Province as an example, 94.42% PCE of AIDS came from the central government and local governments. It showed that the government played a crucial role in the prevention and treatment of AIDS. Compared with some countries and regions that need to rely on social donations, China has a stable financing structure for AIDS prevention and control. According to the Regulations on the Prevention and Control of Infectious Diseases of China, AIDS prevention services are mainly provided by public health institutions, while treatment services are provided by medical institutions. Our results showed that prevention costs mainly go to public health institutions, and treatment costs almost all go to hospitals. It can be seen that the institutional allocation of AIDS prevention and control costs is reasonable.

From the flow of expenditure, the expenditure of AIDS was more spent on prevention than treatment, consistent with the research of Li based on NASA [11]. It also demonstrated that the work of AIDS control in Hunan Province conforms to the principle of “prevention first” that the World Health Organization and China have always adhered to [20]. However, from the use of the preventive expenditure, AIDS prevention in China is still taking a more conservative approach, and some advanced prevention technologies such as pre-exposure prophylaxis (PrEP) for HIV high-risk groups have not yet been applied in clinic. The 2015 world health organization report informed us that clinical trials in Africa, Asia, Europe, South America and the United States have demonstrated that oral PrEP had a high-quality inhibitory effect on the occurrence of HIV infection [21]. Drawing on international experiences, it is suggested to integrate traditional with advanced methods to improve the effectiveness of AIDS prevention by adjusting the allocation of the expenditure of AIDS prevention.

People living with HIV may have to bear part of the cost of services other than antiretroviral treatment. When the funding structure of CCE by age group were compared, the proportion of the family health expenditure were found to be diverse between different age groups. Patients of 40–49 y had the highest proportion of family health expenditure, which may be affected by factors that patients were not insured or co-infected. Of the patients of aged 40–49 y we surveyed, 36% received treatment by self-paying and 48.23% had comorbidity. It is worth noting that the family health expenditure of patients aged 0–9 y exceeds 30%. Children infected with AIDS mainly through mother-to-child transmission. Catastrophic health spending is inevitable if their families have to pay for the treatment of two or more patients. In recent years, the Chinese government has set up special funds to support a series of measures to block mother-to-child transmission of AIDS. Through these measures, such as free AIDS testing and antiviral treatment for infected pregnant women and babies, the mother-to-child transmission rate of AIDS has dropped to 4.9% in 2017 [22]. Simultaneously, the family medical burden problems can not be ignored. It is necessary to bring such families into the scope of government medical assistance while further strengthening measures to block mother-to-child transmission.

Concerning the treatment cost, the result of ordered logistic regression showed that non insurance, co-infection and length of stay were risk factors. Si Cunwu’s research found that by using different payment methods of medical insurance, medical insurance can play a role in controlling the increase of costs for the suppliers and needs of AIDS treatment. Patients who have not moved to medical insurance are not only lack of awareness of cost estimation, but also medical institutions are more likely to have excessive medical practices [23]. It can be seen that incorporating uninsured patients into social basic insurance schemes is the most direct way to reduce the OOPs of patients. Moreover, commercial medical insurance can be encouraged to join the AIDS medical insurance system. With the abundant actuarial data of insurance, we can estimate the risk of infection of high-risk groups and the general population, and integrate AIDS into commercial insurance by adjusting insurance costs. In this way, once people are infected with AIDS, commercial insurance can be used to share the burden of medical treatment.

AIDS patients are vulnerable to pathogens with weaker virulence than healthy people. The resulting infectious diseases are called opportunistic infections of AIDS [24]. Many researches showed that with the opportunistic infection would bring more difficulties and costs for affected people [25, 26]. With regard to the medical security of AIDS patients in opportunistic infections, a series of measures have been introduced in China, including the inclusion of opportunistic infections treatment drugs in the list of essential medicines and the inclusion of AIDS opportunistic infections in the coverage of major diseases in the New Rural Cooperative Medical System. But according to relevant policies, only part of the cost of treatment for opportunistic infections can be reimbursed. In addition, patients with opportunistic infections need to spent more on the treatment, but the reimbursement rate and capping line have not been adjusted. Therefore, in order to reduce the burden of AIDS patients with co-infection, the government should consider to adjust the coverage and proportion of insurance reimbursement appropriately.

With regard to the influence of hospitalization days, Xie claimed that “the longer the length of hospitalization, the higher the cost of hospitalization” [27], which is accordant with our study. Hospitals should control unnecessary large-scale instrument examination under the premise of ensuring medical quality, reduce or eliminate invalid examination items, so as to shorten hospitalization days and alleviate the economic burden of patients. Meanwhile, the early diagnosis and treatment can shorten the length of stay. On the other hand, psychological counseling should be strengthened to avoid the fear caused by the lack of knowledge of AIDS and reduce the avoidable cost of hospitalization due to fear.

Regarding the hospitalization expenses of different age groups, our statistical analysis results showed that the hospitalization expenses of group over 60 y were higher than those in the group under 30 y. There were several reasons to explain this cost gap. The elderly have relatively little awareness of AIDS or AIDS testing [28]. A family survey data indicated that people over 50 y were less likely to have been tested for HIV than those aged 15–49 y [29]. These elderly cannot find out that they have been infected in time unless they have clinical symptoms of AIDS. Once clinical symptoms occur, the treatment becomes more difficult and costly. Meanwhile, the elderly with AIDS are at the high risk of comorbidity [30]. The research of Negin found that the rates of chronic disease were higher among older adults compared with those aged 18–49 y. Of those aged 50 y or more, 29.6% suffered from two or more of the seven chronic conditions compared with 8.8% of those aged 18–49 y [31]. Futhermore, aging AIDS patients may experience a gradual decline in immunity, who are likely to be co-infected. The increasing complexity of patients’ illness necessitates more hospital stay. These may lead to an increase in the cost of treatment for elderly AIDS patients. As for OOPs, patients over 60 y are less than patients under 30 y, which may be the impact of medical insurance compensation and medical assistance policy. According to the study of Xu [32], the proportion of AIDS patients under 40 y of age participating in medical insurance in China is significantly lower than that of people over 60 y. From the medical aid policy, the elderly with no fixed income and no working ability can meet the requirements of applying for medical assistance. So their OOPs is significantly lower than that of other groups.

To our knowledge, this study is the first one to give a comprehensive report about expenditures of AIDS in China at the subnational level based on SHA2011. In this study, not only the scale and flow of PCE for AIDS were calculated from a macro point of view, but also the factors affecting the medical burden of AIDS patients were statistically analyzed from a micro point of view. However, it is undeniable that the study has two main limitations. Compared with the actual situation, the AIDS preventive expenditure based on the SHA2011 framework could be underestimated, because the condoms at patients’ own expense was not taken into account. In addition, in the calculation of CCE, only considered direct medical expenses were considered, excluding indirect expenses (such as transportation fee and loss of medical working hours, etc). Therefore, our calculation of OOPs could not fully reflect the actual economic burden of AIDS families.

## Conclusions

The PCE for AIDS based on SHA2011 can not only reveal the overall situation of AIDS PCE, but also analyze the financing burden of different groups. The PCE for AIDS in Hunan Province was 266.67 million in 2017, mainly used for prevention. The financing structure was stable and the flow of institutions was reasonable. Nevertheless, there were still some groups with the risk of poverty caused by AIDS treatment. Combined with the orderly logistic regression analyses, it was suggested that the burden of AIDS patients be reduced by optimizing the allocation of prevention costs to increase investment in efficient prevention methods, expanding the scope of government assistance, adjusting insurance compensation measures, increasing the rate of patients participating in social basic insurance, and controlling length of stay in hospital. Moreover, the commercial medical insurance should be incorporated into the AIDS medical insurance system to share the patients’ burden of medical treatment.

## Data Availability

The datasets generated and/or analyzed during the current study are available from the corresponding author on reasonable request.

## Abbreviations

CCE: Curative care expenditure
OOPs: Out-of-pocket payments
PCE: Preventive and curative care expenditure
SHA 2011: System of Health Accounts 2011
Totalexp: the total hospitalization expenses
